# Elution kinetics of vancomycin and gentamicin from carriers and their effects on mesenchymal stem cell proliferation: an in vitro study

**DOI:** 10.1186/s12891-017-1737-4

**Published:** 2017-09-02

**Authors:** Tomas Kucera, Lenka Ryskova, Tomas Soukup, Jana Malakova, Eva Cermakova, Pavel Mericka, Jakub Suchanek, Pavel Sponer

**Affiliations:** 1 Department of Orthopaedic Surgery, University Hospital Hradec Kralove, Charles University in Prague, Faculty of Medicine in Hradec Kralove, Sokolska 581, 500 05 Hradec Kralove, Czech Republic; 2 Department of Clinical Microbiology, University Hospital Hradec Kralove, Charles University in Prague, Faculty of Medicine in Hradec Kralove, Sokolska 581, 500 05 Hradec Kralove, Czech Republic; 3 Department of Histology and Embryology, Charles University in Prague, Faculty of Medicine in Hradec Kralove, Sokolska 581, 500 05 Hradec Kralove, Czech Republic; 4 Department of Clinical Biochemistry, University Hospital Hradec Kralove, Charles University in Prague, Faculty of Medicine in Hradec Kralove, Sokolska 581, 500 05 Hradec Kralove, Czech Republic; 5 Department of Medical Biophysics, Charles University in Prague, Faculty of Medicine in Hradec Kralove, Sokolska 581, 500 05 Hradec Kralove, Czech Republic; 6 Tissue Bank, University Hospital Hradec Kralove, Sokolska 581, 500 05 Hradec Kralove, Czech Republic; 7 Department of Dentistry, University Hospital Hradec Kralove, Charles University in Prague, Faculty of Medicine in Hradec Kralove, Sokolska 581, 500 05 Hradec Kralove, Czech Republic

**Keywords:** Local antibiotic carriers, Stem cells, Musculoskeletal infections

## Abstract

**Background:**

Musculoskeletal infections remain a major complication in orthopedic surgery. The local delivery of antibiotics provides the high levels required to treat an infection without systemic toxicity. However, the local toxicity of antibiotic carriers to the mesenchymal stem cells, as a result of both the peak concentrations and the type of carrier, may be significant.

**Methods:**

To address this concern, the elution kinetics of vancomycin and gentamicin from several commercially available antibiotic carriers and several carriers impregnated by a surgeon (10 ml of each sterile carrier were manually mixed with a 500 mg vancomycin and an 80 mg gentamicin solution, and the duration of impregnation was 30 min) were assessed. Moreover, the effects of these antibiotic carriers on stem cell proliferation were investigated. The following two types of stem cells were used: bone marrow and dental pulp stem cells.

**Results:**

The high eluted initial concentrations from antibiotic impregnated cancellous allogeneic bone grafts (which may be increased with the addition of fibrin glue) did not adversely affect stem cell proliferation. Moreover, an increased dental pulp stem cell proliferation rate in the presence of antibiotics was identified. In contrast to allogeneic bone grafts, a significant amount of antibiotics remained in the cement. Despite the favorable elution kinetics, the calcium carriers, bovine collagen carrier and freeze-dried bone exhibited decreased stem cell proliferation activity even in lower antibiotic concentrations compared with an allogeneic graft.

**Conclusions:**

This study demonstrated the benefits of antibiotic impregnated cancellous allogeneic bone grafts versus other carriers.

## Background

In a large cohort study of the Norwegian Arthroplasty Register, it was shown that the local delivery of antibiotics from bone cement combined with systemic antibiotics had the lowest risk of revision after 0 – 14 years [1]. In contrast, bone cement with antibiotics acts as a permanent foreign body, which may be colonized by bacteria. Moreover, the elution of sub-inhibitory antibiotic levels over an extended period may induce bacterial resistance [2]. Neut et al. assessed gentamicin-loaded cement beads removed at the time of the second stage of a two-stage procedure for infection and identified the presence of bacteria on these beads in 18 of 20 cases; moreover, 19 of the 28 bacterial strains isolated in this study were resistant to gentamicin [3]. To overcome the problem regarding the permanent presence of cement as a foreign body, degradable local antibiotic carriers or bone grafts, which may simultaneously eradicate pathogens and facilitate bone regeneration, and the biological incorporation of a new implant have been developed. Winkler et al. have used freeze-dried bone, and the grafts were incubated in an antibiotic solution for 24 h [4]. A perioperative impregnation of carriers with antibiotics selected for a specific situation in a specific patient may be preferable; however, as a weakness, it may be associated with unpredictable pharmacokinetics. In general, released antibiotics must reach concentration levels above the minimal bactericidal concentration to prevent bacterial resistance; however, the peak concentration must also not affect bone healing. Bone healing may be affected by many other factors, including the type of the carrier. Furthermore, various types of mesenchymal stem cells may differentially respond to antibiotic impregnated carriers. Previous studies have predominately investigated aminoglycosides and glycopeptides in local antibiotic carriers because of their good pharmacokinetic properties and susceptibility to causative pathogens in implant surgery [4, 5]. Despite the fact that monoantibiotic–loading is described in most cases, a synergistic effect has been described between aminoglycosides and glycopeptides when eluted from acrylic bone cement [6]. In our clinical practice, gentamicin alone was effective in 64.2% compared with 89.8% for vancomycin, whereas the combination of vancomycin and gentamicin in 96.4% of causative pathogens delayed periprosthetic infections.

The aims of this study were to analyze the elution kinetics of vancomycin and gentamicin from carriers impregnated with antibiotics by a surgeon and commercially available antibiotic carriers. The carriers were impregnated with both antibiotics. Moreover, the effects of these antibiotic carriers (effects of the released antibiotic concentration and carrier properties together) on stem cell proliferation were investigated. The following two types of stem cells were used: bone marrow and dental pulp stem cells.

## Methods

### Selection of carriers and impregnation

Four carriers for antibiotic impregnation that may facilitate bone regeneration and three commercially available antibiotic-loaded carriers as a control group were selected.

#### Control group:

1. VancogenX (Tecres S.p.a., Italy) – polymethylmethacrylate nonresorbable bone cement that contained gentamicin sulfate and vancomycin hydrochloride (equivalent to a 1 g gentamicin base and a 1 g vancomycin base in a 40 g unit). The powder and liquid were mixed, and 1 mL volume cubes were created.
2. Septocoll® E (Biomet, Germany) - resorbable, equine collagen fleece, which included 5 × 4 × 0.3 cm fleece that contained 80 mg collagen (equine), 29 mg gentamicin sulfate (equivalent to 17.5 mg gentamicin) and 87 mg gentamicin crobefate (equivalent to 17.5 mg gentamicin). The carrier was morselized into small pieces.
3. Herafill® beads G (Heraeus, Germany) – resorbable beads (4 beads/1 mL) that contained calcium sulfate dihydrate, calcium carbonate, hydrogenated triglyceride, and gentamicin sulfate (2.5 mg of gentamicin/bead).

#### Carriers for impregnation:

1. 1/ Allogeneic cancellous bone from the proximal tibia (Tissue Bank of our University Hospital), which was morselized into small pieces.
2. 2/ The same carrier mixed with fibrin glue (Tissucol, Baxter, Nederland), four milliliters of Tissucol were added to 10 mL of morselized cancellous bone.
3. 3/ Freeze-dried bone in powder (National Tissue Centre).
4. 4/ Tricalcium phosphate with 90% porosity (Vitoss bone graft substitute, Orthovita, USA), morselized into small pieces.

Ten milliliters of each sterile carrier were manually mixed with an antibiotic-containing solution (500 mg of vancomycin and 80 mg of gentamicin), and the duration of impregnation was 30 min. The time of preparation was considered acceptable in the course of the operation for impregnation. Edicin 0.5 g/10 mL solution (vancomycin hydrochloridum) (Lek Pharmaceuticals d.d., Slovenia) and Gentamicin 80 mg/2 mL solution (gentamicin sulfate) (Lek Pharmaceuticals d.d., Slovenia) were used. The antibiotic dose was chosen to ensure the final concentrations were comparable to the concentrations in the control group.

After impregnation, three samples from each carrier were randomly obtained to investigate the homogeneity; the volume of each sample was 1 mL (21 samples of 1 mL from 7 carriers). Each 1 mL of antibiotic impregnated carrier was made up to 10 mL with Phosphate Buffered Saline (Gibco, United Kingdom), and all samples were placed in a thermostat and incubated at 37 °C in a 5% CO_2_ atmosphere.

Assuming a uniform impregnation of the tested carriers, every 1 mL of impregnated carriers contained 8 mg of gentamicin and 50 mg of vancomycin. The control group contained 1 mL of Septocoll® E – 5.8 mg of gentamicin, 1 mL of Herafill® beads G – 10 mg of gentamicin and 1 mL of VancogenX – 41 mg of vancomycin and 41.2 mg of gentamicin.

### Measurement of antibiotic concentrations

The entire amount of Phosphate Buffered Saline was exchanged daily. The antibiotic concentration in the solution was measured on the 1st, 2nd, 3rd, 4th, 7th, 10th, 14th, 17th, 21st and 25th days after impregnation immediately following the Phosphate Buffered Saline exchange. A quantitative determination of antibiotics in the samples from the experiments was performed using a fluorescence polarization immunoassay in an Integra 400 Plus analyzer (Roche Diagnostics GmbH, Mannheim, Germany). The measurements were performed following the manufacturer’s instructions for the reagents. During the measurement of the antibiotic concentration, the analytical method provided the concentration of the internal quality control samples in the required range. The inter-batch precision expressed as the coefficient of variation ranged from 4.84% to 7.18% for gentamicin compared with 3.53% to 5.98% for vancomycin. The intra-batch precision ranged from 1.17% to 2.13% for gentamicin compared with 1.11% to 1.16% for vancomycin.

### Bone Marrow Mesenchymal Stem Cells (BM MSC) and Dental Pulp Stem Cells (DPSC)

Bone marrow (BM) was obtained from 2 donors without any comorbidities undergoing a total hip replacement due to osteoarthritis following informed consent according to University Hospital guidelines. Dental pulp stem cells were isolated from impacted third molars obtained from 2 healthy donors undergoing tooth extraction for orthodontic reasons. The subjects or their parents/guardians provided written informed consent for the use of the extracted teeth following ethical approval by the Ethics Committee of the Medical Faculty. Patients suffering from cancer or infectious diseases or patients undergoing dialysis or immunosuppression were excluded from our study.

Both types of isolated fully characterized stem cells were treated with standard operation protocols as previously described [7–9].

### Culture conditions

Cells were cultured on untreated plastic (TPP Petri-dishes and TPP Multi-Well Plates) at 37 °C under aerobic conditions (5% CO2) with a 2% Fetal calf serum - containing alpha-MEM expansion medium. The optimized mesenchymal stem cells (MSC) expansion medium [10] consisted of standard cultivation media composed of α-MEM, 2% Fetal calf serum, 10 ng/ml endothelial growth factor (PeproTech, London, UK), 10 ng/ml platelets derived growth factor (PeproTech), L-ascorbic acid 1% (Sigma), 2% glutamine (Invitrogen), penicillin/streptomycin 0,5% (Invitrogen), gentamicin 0,5% (Invitrogen), dexamethasone 8% (Sigma) and was supplemented with insulin-transferrin-sodium-selenite supplement (Sigma) at a concentration of 10 μl/ml. Once the adherent cells were more than 70% confluent, they were detached with 0.25% trypsin-EDTA (Invitrogen), counted (using a Z2 counter (Beckman Coulter, USA)) and replated at a 1:3 dilution under the same culture conditions.

For our experiment, the cells (passage No. 3) were plated into 12-well multidishes (Corning, USA), which included 50,000 cells per well. Following 24 h of cultivation, Transwell Inserts /24 mm in diameter, pores 0.4 μm, and a polyester membrane (Corning) were added to each well. Two mL of fresh cultivation media were added to ensure a total volume of 4 mL. The previously described carriers were subsequently added to the cultivation system without direct contact of the carriers with the MSC (1. allogeneic cancellous bone without antibiotics, 2. allogeneic cancellous bone with antibiotics, 3. allogeneic cancellous bone mixed with fibrin glue without antibiotics, 4. allogeneic cancellous bone mixed with fibrin glue with antibiotics, 5. tricalcium phosphate without antibiotics, 6. tricalcium phosphate with antibiotics, 7. freeze-dried bone without antibiotics, 8. freeze-dried bone with antibiotics, 9. Herafill® beads G (Heraeus, Germany), 10. Septocoll® E (Biomet, Germany), 11. bone cement VancogenX (Tecres S.p.a., Italy), 12. control group – MSC without carrier). The cells were cultivated for 14 days. Analyses of the proliferation were performed using a Z2 counter (Beckman Coulter, USA) as previously described. The cultivation medium was changed daily and mixed in the culture system twice daily.

### Statistical analysis

All experiments were conducted in duplicate.

The program NCSS 9, Statistica 12 was used for the statistical analysis as follows: descriptive statistics (mean, standard deviation, standard error, minimum, maximum, range, and quartiles) were performed, including box plots, repeated measures analysis of variance with multiple comparisons by Fisher’s LSD test, and a Kruskal – Wallis nonparametric analysis of variance with multiple comparisons by Dunn’s test. The level of significance was α = 0.05.

## Results

### In vitro assessment of antibiotic elution kinetics

**Fig. 1:**
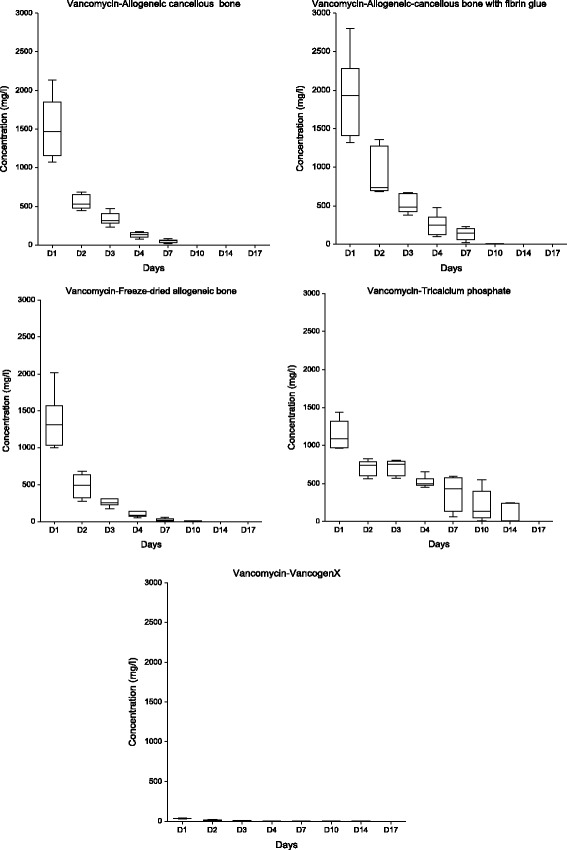
Elution kinetics of vancomycin from the impregnated carriers and commercially available bone cement: Box plot. Data represent the means (+SD) of duplicate experiments. Significant differences are described in the results text (repeated measures analysis of variance with multiple comparisons, ANOVA, the level of significance was α = 0.05)

**Fig. 2:**
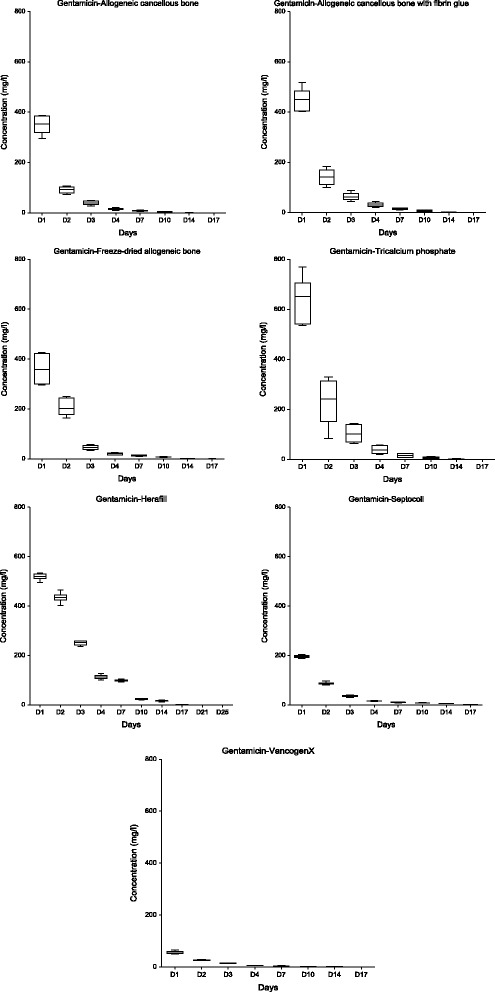
Elution kinetics of gentamicin from the impregnated carriers and commercially available bone cement, collagen carrier and calcium sulfate dihydrate/calcium carbonate carrier: Box plot. Data represent the means (+SD) of duplicate experiments. Significant differences are described in the results text (repeated measures analysis of variance with multiple comparisons, ANOVA, the level of significance was α = 0.05)

The elution kinetics of the antibiotics from the carriers are presented in box plots (Figs. 1 and 2).

#### Antibiotic laden bone cement (VancogenX)

The cement generated the lowest initial mean concentrations of both antibiotics. The final measurable concentrations were obtained on the 17th day. Long-term elution was not confirmed.

#### Septocoll®

This collagen gentamicin carrier generated increased concentrations for a longer time period compared with the bone cement.

#### Herafill® beads G

This resorbable gentamicin carrier exhibited significantly different elution kinetics until the seventh day compared with the bone cement (α < 0.05). The characteristics were similar to impregnated carriers.

#### Allogeneic cancellous bone

This impregnated carrier generated substantially increased initial antibiotic concentrations with significant differences in the elution kinetics only in the first 3 days (α < 0.05); there was a shorter elution time of vancomycin and the same elution time in the case of gentamicin compared with the bone cement.

#### Allogeneic cancellous bone with fibrin glue

The mixture with a fibrin glue resulted in even higher antibiotic concentrations with the same characteristics as the allogeneic cancellous bone alone. The addition of the fibrin glue did not contribute to the prolongation of the elution period.

#### Freeze-dried allogeneic bone

This carrier exhibited similar elution kinetics as the allogeneic cancellous bone without significant differences in the assessments (α > 0.05); moreover, it had the same relationship to bone cement. The hypothesis regarding the absorption of substantially increased quantities of aqueous solution during rehydration by a carrier in a dry state was not confirmed.

#### Tricalcium phosphate

Significant differences in the elution kinetics of vancomycin with a slower decrease in the last measurable values on the 14th day were identified compared with the bone cement and all other carriers in the assessments (α < 0.05). This carrier exhibited the highest 1st day gentamicin concentration with similar elution kinetics compared with previous carriers.

**Fig. 3:**
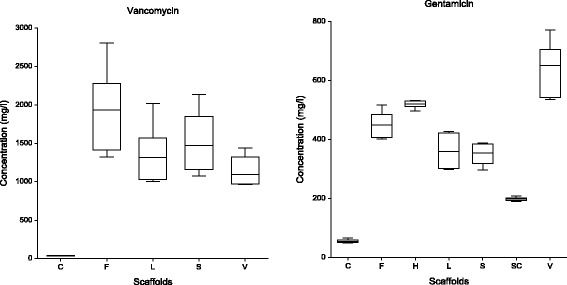
Total amount of measured vancomycin and gentamicin concentrations: Box plot. C – cement, F – allogeneic cancellous bone with fibrin glue, L – freeze-dried allogeneic bone, S - allogeneic cancellous bone, V – tricalcium phosphate, H - Herafill® beads, SC - Septocoll®. Data represent the means (+SD) of duplicate experiments. Significant differences are described in the results text (repeated measures analysis of variance with multiple comparisons, ANOVA, the level of significance was α = 0.05)

The total amounts of measured antibiotic concentrations are indicated in Fig. 3.

The total amount of measured vancomycin concentrations indicated the lowest values in the bone cement (C), statistically comparable values in the allogeneic cancellous bone (S), freeze-dried bone (L) and tricalcium phosphate (V) and the highest values in the allogeneic cancellous bone with fibrin glue (F). The total amounts of measured gentamicin concentrations exhibited significantly different values: the lowest concentration in cement (C), followed by Septocoll® (SC), with comparable values in the allogeneic cancellous bone (S) and freeze-dried bone (L), comparable values in the allogeneic cancellous bone with fibrin glue (F) and Herafill® beads G (H) and the highest concentration in tricalcium phosphate (V).

### Effects of carriers on BM MSC and DPSC proliferation

**Fig. 4:**
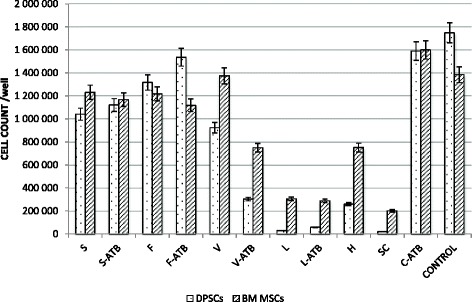
BM MSC and DPSC count (y-axis) following the cultivation of 50,000 stem cells per well for 14 days in the presence of carriers: S - allogeneic cancellous bone, S-ATB - allogeneic cancellous bone with vancomycin and gentamicin, F – allogeneic cancellous bone with fibrin glue, F-ATB - allogeneic cancellous bone with fibrin glue with vancomycin and gentamicin, V – tricalcium phosphate, V-ATB - tricalcium phosphate with vancomycin and gentamicin, L – freeze-dried allogeneic bone, L-ATB – freeze-dried allogeneic bone with vancomycin and gentamicin, H - Herafill® beads with gentamicin, SC - Septocoll® with gentamicin, C – cement with vancomycin and gentamicin, CONTROL – without antibiotics or a carrier

The proliferation of bone marrow mesenchymal stem cells in the presence of carriers with or without antibiotics is presented in Fig. 4. The proliferation activity was comparable to the control group (CONTROL) in the bone cement (C-ATB), in the allogeneic cancellous bone and allogeneic cancellous bone with fibrin glue (both with and without antibiotics: S, S-ATB, F, F-ATB). A high initial concentration of antibiotics in cancellous bone did not significantly affect the MSC proliferation activity, with equally low concentrations in the bone cement. A significant difference was identified in the case of tricalcium phosphate: a good proliferation activity was identified in the carrier without antibiotics (V) and distinctly lower activity in the presence of antibiotics (V-ATB), which was comparable with another calcium carrier, Herafill® beads G (H). Freeze-dried allogeneic bone (L and L-ATB) and Septocoll® E (SC) that contained equine collagen had the most pronounced negative effect on the MSC proliferation. A difference in the proliferation activity between the BM MSC and DPSC was identified: a decreased proliferation rate of BM MSC in all carriers with antibiotics compared with the carriers without antibiotics and an increased proliferation activity of DPSC cultivated with antibiotic loaded allogeneic cancellous bone (both with and without fibrin glue) and with freeze-dried bone. The significant differences in the cell count per well between BM MSC and DPSC excluding the bone cement and the allogeneic cancellous bone with antibiotics were found (α < 0.05).

## Discussion

Degradable local antibiotic carriers or bone grafts may simultaneously eradicate pathogens and facilitate bone regeneration and there is the increasing interest in high-concentration local antibiotic delivery systems in the management of the musculoskeletal infections. An appropriate local antibiotic delivery system depends on the pharmacokinetic properties. These properties are affected besides by the antibiotic choice, impregnation methods, exact antibiotic/carrier ratio. An apparent difference between the initial amount of antibiotics for the impregnation and the low values of their concentrations in the supernatans may be present. In addition, the high concentrations of antibiotics may be toxic for cells (cell death or cells that survive are not osteogenic). In this study, we were interested in the effects of antibiotics and carriers on the stem cells niche and the changes of the proliferative potential. We are aware that the bone repair is complex multifactorial process that also involves angiogenesis, cell migration and a variety of cell types. The effects of antibiotics and their carriers on bone healing must be investigated on in vivo model.

Previous studies have demonstrated that antibiotics elute from an antibiotic laden cement over a 6–8 week period [5]; however, the last measurable (even sub-inhibitory) concentrations were identified on the 17th day with the lowest eluted amount of antibiotics in our study. This finding supports the theory that antibiotics are mainly released by a surface phenomenon, and a substantial amount remains encapsulated in bone cement [11]. Powles et al. reported that the fracture of the cement mantle may liberate substantial levels of antibiotics years after the original procedure was performed [12]. A sub-inhibitory concentration significantly increases the risk of bacterial resistance induction.

The reported drawbacks of collagen carriers include non-matching, long degradation rates and short release times (collagen may serve as a substrate for bacterial adhesion), complicated handling, and biological responses, such as localized hypersensitivity and the circulation of antibodies against bovine collagen [13]. In our study, the lowest amount of harvested cells was obtained from the samples that contained Septocoll. Septocoll is composed of equine collagen, and the matrix creates a membrane-like structure. Following the insertion of Septocoll in the cultivation media, the Septocoll spread and covered the surface of the cultivation flask. We assume that this process leads to low oxygen perfusion, hypoxia and the ultimate death of cultivated cells. This carrier exhibited the lowest biocompatibility in our study, and we do not use it in our clinical practice. Formerly, we used Septocoll in the surgical treatment of musculoskeletal infections and we recorded frequent secretion from the surgical wound.

Resorbable beads that contain calcium sulfate/calcium carbonate with gentamicin sulfate exhibited a longer elution time with increased antibiotic concentrations compared with bone cement. The lower BM MSC proliferation activity in the presence of this carrier was its drawback. To enhance its biocompatibility, it is mixed with bone grafts in our clinical practice. Similar experiences have been reported by Coraca-Huber et al. [14]. Moreover, a significantly lower proliferation rate was identified in DPSC.

Tricalcium phosphate exhibited favorable elution kinetics for both antibiotics, which may be a result of the very high porosity of this carrier (90%). Studies have indicated the advantageous elution kinetics of antibiotics from different calcium-phosphate carriers [15]; nevertheless, similar to the calcium sulfate/calcium carbonate in our study, a decreased MSC proliferation rate was identified in the presence of antibiotics.

Winkler et al. have used freeze-dried bone, and grafts were incubated in an antibiotic solution for 24 h; the vancomycin and tobramycin concentrations in the human bone remained above the minimum inhibitory concentration for at least 13 days with completely replaced medium every 24 h for 10 days, followed by replacement on days 13, 15, 20, 22 and 28 [4]. The authors used impregnated grafts with vancomycin and tobramycin in clinical practice during a one-stage revision of a periprosthetic joint infection with encouraging results [16]. However, in our study, the comparable elution time was achieved following impregnation in an antibiotic solution for 30 min; this approach enables a perioperative preparation of carriers with antibiotics selected for a specific situation in a specific patient and is significantly cheaper.

Furthermore, the cells cultivated with freeze-dried allogeneic bone exhibited a decreased proliferation rate compared with both other allogeneic bone groups independent of antibiotics. Cornu demonstrated that freeze drying and irradiation lead to increased brittleness and indicated that the destruction of the organic matrix is one potential reason for this change [17].

Allogeneic cancellous bone with or without fibrin glue exhibited a comparable elution time of antibiotics with bone cement (the same in the case of gentamicin, shorter in vancomycin). Studies have indicated a rapid release in the first 2 days depending on the gentamicin concentration mixed with demineralized bone [18]. However, high antibiotic concentrations in the first days in our study did not have a negative effect on MSC proliferation. Rathbone et al. reported that vancomycin may be released even at concentrations up to 2000 mg/L with a decrease of less than 25% in the alkaline phosphatase activity and DNA content in osteoblast cells, whereas gentamicin concentrations between 10 and 200 mg/L caused a decrease in the alkaline phosphatase activity and DNA content of 25% - 50% [19]. We measured the proliferation activity of MSC after 14 days; thus, we suggest that short-term exposure to high antibiotic concentrations in the initial days does not adversely permanently affect this activity.

A decreased proliferation rate of BM MSCs cultivated with the scaffold loaded with antibiotics compared with the proliferation rate of cells cultivated without antibiotics was expected; however, we cannot fully explain why the DPSC reacted in the opposite manner when they were cultivated together with the allogeneic bone, allogeneic bone with fibrin glue and freeze-dried bone. The DPSC exhibited an even higher proliferation activity in the presence of high concentrations of antibiotics.

Several weaknesses of this study must also be considered. Kühn compared the release of 12 different bone cements in vitro over 7 days and identified a difference in the release of gentamicin [20]. Moreover, Meyer et al. demonstrated the effect of cement vacuum-mixing on antibiotic elution characteristics [21]. Only one type of bone cement was implemented in our study. As a result of the spectrum of causative pathogens of musculoskeletal infections in our clinical practice, we were forced to include vancomycin for local therapy, and VancogenX was the only available cement that contained vancomycin. Furthermore, the main problem of all in vitro studies was present, i.e., their capability to predict the real clinical performance in human patients. In general, antibiotic doses and impregnation methods could be clearly defined in vitro and the appropriate pharmacokinetics could be demonstrated; however, it is critical to understand the methods of a study as follows: the release time of antibiotics predominately depends on the frequency of the measurement and the amount of exchanged medium. Bormann et al. mixed vancomycin, gentamicin and tobramycin with a demineralized bone matrix or sodium hyaluronate in a special mixing device. In the case of the 100% medium exchange (similar to our study), the release of gentamicin and tobramycin was complete after 3 days and vancomycin was completely released after 14 days; in the case of less frequent sampling with a 50% medium exchange, there was inhibited growth of *Staphylococcus aureus* for up to 56 days [22]. However, the crucial factors include the in vivo stability and clearance of antibiotics in specific patients, which may be difficult to predict. We are preparing an in vivo study to confirm our in vitro results.

## Conclusions

In summary, this study demonstrated the benefits of antibiotic impregnated cancellous allogeneic bone grafts versus other carriers, including antibiotic laden cement, as follows: a rapid elution of antibiotics from the grafts was excluded, even when gentamicin had the same elution time in both the grafts and cement; the high eluted initial concentrations from grafts (which may be increased with the addition of fibrin glue) did not adversely affect the BM MSC or DPSC proliferation. Moreover, an increased DPSC proliferation rate in the presence of antibiotics was identified. In contrast to allogeneic bone grafts, a significant amount of antibiotics remained in the cement and the cement generated the lowest initial mean concentrations of both antibiotics. Despite favorable elution kinetics, in the calcium carriers, bovine collagen carrier and freeze-dried bone, which are widely used as local antibiotic carriers, decreased BM MSC and DPSC proliferation activities were identified even in lower antibiotic concentrations compared with the allogeneic graft.

## Abbreviations

BM MSC: Bone marrow mesenchymal stem cells
BM: Bone marrow
C, C-ATB: Antibiotic-loaded cement with vancomycin and gentamicin
CONTROL: Without antibiotics or a carrier
DPSC: Dental pulp stem cells
F: Allogeneic cancellous bone with fibrin glue
F-ATB: Allogeneic cancellous bone with fibrin glue with vancomycin and gentamicin
H: Herafill® beads with gentamicin
L: Freeze-dried allogeneic bone
L-ATB: Freeze-dried allogeneic bone with vancomycin and gentamicin
MSC: mesenchymal stem cells
S: Allogeneic cancellous bone
S-ATB: Allogeneic cancellous bone with vancomycin and gentamicin
SC: Septocoll® with gentamicin
V: Tricalcium phosphate
V-ATB: Tricalcium phosphate with vancomycin and gentamicin
